# Controlling the frequency dynamics of homing gene drives for intermediate outcomes

**DOI:** 10.1093/g3journal/jkae300

**Published:** 2024-12-19

**Authors:** Benjamin J Camm, Alexandre Fournier-Level

**Affiliations:** School of BioSciences, The University of Melbourne, Parkville, VIC 3010, Australia; School of BioSciences, The University of Melbourne, Parkville, VIC 3010, Australia

**Keywords:** CRISPR, modeling, simulation, conversion efficiency, selection, inbreeding, dominance, resistance, fitness cost

## Abstract

Gene drives have enormous potential for solving biological issues by forcing the spread of desired alleles through populations. However, to safeguard from the potentially irreversible consequences on natural populations, gene drives with intermediate outcomes that neither fixate nor get removed from the population are of outstanding interest. To elucidate the conditions leading to intermediate gene drive outcomes, a stochastic, individual allele-focused gene drive model was developed to simulate the diffusion of a homing gene drive in a population. The frequencies of multiple alleles at a locus targeted by a gene drive were tracked under various scenarios. These explored the effect of gene drive conversion efficiency, strength and frequency of resistance alleles, dominance and strength of a fitness cost for the gene drive, and the level of inbreeding. Four outcomes were consistently observed: fixation, loss, temporary, and equilibrium. The latter 2 are defined by the frequency of the gene drive peaking then crashing or plateauing, respectively. No single variable determined the outcome of a drive. The difference between the conversion efficiency and resistance level, modeled quantitatively, differentiated the temporary and equilibrium outcomes. The frequency dynamics of the gene drive within outcomes varied extensively, with different variables driving these dynamics between outcomes. These simulation results highlight the possibility of fine-tuning gene drive outcomes and frequency dynamics. To that end, we provide a web application implementing our model, which will guide the safer design of gene drives able to achieve a range of controllable outcomes tailored to population management needs.

## Introduction

Gene drives are a promising, yet contentious, avenue of genetic biocontrol at our disposal to rectify pressing ecological issues. A gene drive can be used to alter, replace, or suppress a population by spreading a desired allele through segregation distortion and particularly super-Mendelian inheritance (Feldman and Otto 1991; Gantz and Bier 2016; McFarlane *et al*. 2018; Rode *et al*. 2019). While gene drives have powerful features, they are equally hazardous. Gene drives can spread beyond their intended range (Greenbaum *et al*. 2021) or act in unintended ways (DiCarlo *et al*. 2015), potentially with detrimental consequences for the environment (Burt 2003). As such, modeling is a prudent approach to understand the key features determining gene drives’ behavior in a population before we can be confident that any synthetic gene drive will spread as intended (Combs *et al*. 2023). In particular, high confidence that a particular gene drive design will lead to the expected outcome is central (Prowse *et al*. 2017; James *et al*. 2020; Devos *et al*. 2022).

Previous models have explored how variation in pairs of variables can change either the outcome (Dhole *et al*. 2018; Rode *et al*. 2019) or the frequency dynamics (Unckless *et al*. 2015; Drury *et al*. 2017; Dhole *et al*. 2020) of a gene drive. Most commonly, it is the interaction between conversion efficiency and fitness cost that is explored (Unckless *et al*. 2015, 2017; Drury *et al*. 2017). However, many of the features affecting a gene drive vary continuously, potentially interacting with each other to greatly complicate the outcome predictability (Nash *et al*. 2019; Frieß *et al*. 2023). Other factors like resistance level and frequency will likely further influence a gene drive. Understanding how the many variables affecting a gene drive influence its fate thus requires their simultaneous testing over a continuous range of values. This would provide a more specific depiction of the effect each variable has on the frequency dynamics of a gene drive and finally its outcome.

Four distinct outcomes have been described for gene drives: the fixation of the gene drive allele, the loss of the gene drive allele (Gantz and Bier 2016), the temporary establishment of the gene drive allele (Noble *et al*. 2017; Drury *et al*. 2017; Unckless *et al*. 2017) or the gene drive allele frequency reaching an equilibrium within the population (Unckless *et al*. 2015; Rode *et al*. 2019). The intermediate outcomes, either temporary or equilibrium, are often overlooked in gene drive models in favor of the more extreme fixation or loss outcomes when intending to suppress a population (Backus and Gross 2016; Noble *et al*. 2017; Paril and Phillips 2022). However, these intermediate outcomes could be of great relevance under specific scenarios of population management. It may be safer to design a gene drive that is temporary or reaches equilibrium in a population while the mechanics of the system are not yet fully controlled. A gene drive intended to suppress a local population may have unforeseen consequences that outweigh the potential benefits (Frieß *et al*. 2023). Designing gene drives that are locally confined over time and space are ideal candidates for initial gene drive releases (Dhole *et al*. 2018, 2020; Marshall and Akbari 2018).

The most commonly envisioned means to achieve confinement for a gene drive relies on geographic isolation, which requires the presence of reproductive barriers between target and nontarget populations (Dhole *et al*. 2018; Greenbaum *et al*. 2021; Harris and Greenbaum 2023). To ensure effective spatial containment, continued monitoring would need to be in place to detect any escaped gene drives, which is costly and difficult to implement (Giese *et al*. 2020). Alternatively, having a gene drive reach equilibrium in a population will avoid irreversible loss of alleles, and potentially the whole species, and maintain genetic diversity at the gene drive target locus. Given the inherent relevance of these intermediate gene drive outcomes, particularly in the short term, we need to understand which properties of a gene drive and the target population determine the outcome of a release, and how these properties affect the frequency dynamics over time.

Gene drive modeling has primarily investigated the end point of a gene drive (Unckless *et al*. 2015; Drury *et al*. 2017; Rode *et al*. 2019); mostly asking whether the gene drive would fixate or be lost. However, this also overlooks the many ways a gene drive can reach a given end point. If a gene drive fixates, does it fixate quickly or slowly? If a gene drive temporarily reaches a maximum frequency, what is this maximum frequency? And then does the frequency remain stable or decrease? Understanding how the properties of a gene drive influence its dynamics will greatly increase the likelihood of meeting a desired outcome based on both population and biotechnological features. Furthermore, interactions between the properties of the gene drive and the target population could amplify or reduce their effects, and understanding how these properties interact as a complex system to influence the gene drive dynamics is crucial for safe gene drive design.

A range of gene drive properties have been tested as variables in models, from gene drive design (Dhole *et al*. 2018; Noble *et al*. 2019; Oberhofer *et al*. 2019), environmental effects (North *et al*. 2019) to organism biology (Sánchez *et al*. 2020). The effect of controllable variables, alone or in conjunction, on the frequency dynamics and outcome of a gene drive could be tuned by design or by management to achieve the desired results, potentially compensating for imponderable features of the biological system targeted. Gene drive risk assessment should be exhaustive across ecological contexts (Kim *et al*. 2023) as there will be no risk taken on large-scale exploratory trials (Baltzegar *et al*. 2018; Frieß *et al*. 2023). As the technology is almost at hand, the challenge is to measure the importance of every feature to deliver eco-evolutionary relevant predictions for a gene drive release (Combs *et al*. 2023).

Our study intended to describe the variable space that leads to intermediate gene drive outcomes to guide safer gene drive design (Marshall and Akbari 2018). By running simulations across a large variable space, we have narrowed down the combination of variables that lead to each outcome and determined how these variables influence the frequency dynamics of the gene drive within a given outcome. Through this, we aim to better inform gene drive design to maximize the likelihood that a gene drive will safely and surely meet its intended aim.

## Methods

### Modeling framework

**Fig. 1.**
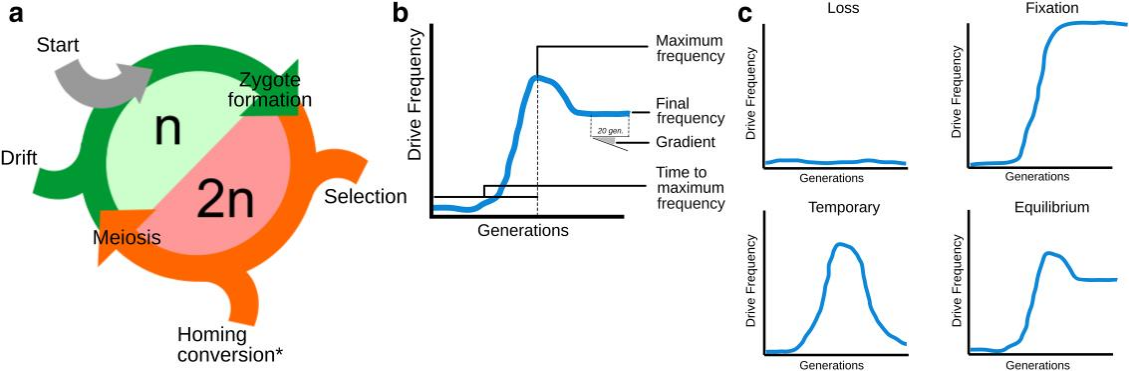
a) Model structure. The model is presented in the case of a prezygotic gene drive, post-zygotic gene drives can be modeled by swapping the homing conversion and selection modules. b) Summary statistics used to define the categorical outcome of a gene drive release. c) Example of frequency dynamics for each of the 4 outcomes.

A stochastic, allele-level CRISPR-based homing gene drive model implemented in the Julia programming language was developed to test the effect of homing conversion efficiency, resistance level, resistance frequency, fitness cost (selection acting against the gene drive), exposure to selection, dominance of the gene drive fitness cost, and inbreeding (Table 1) on the frequency of a gene drive allele, starting with an initial allele frequency of 0.001 and tracked over time in a population. The model cycles through discrete, nonoverlapping generations, running through modules that simulate the life cycle of a sexually reproducing organism with discrete and nonoverlapping transitions between a prezygotic haploid and a post-zygotic diploid stage (Fig. 1a). This modular implementation offers flexibility for modeling different biologies, for example, exploring pre- or post-zygotic acting gene drives by inverting the homing conversion module and the selection module. In prezygotic homing gene drives, homing occurs in the premeiotic diploid cells in the early stages of gametogenesis before the haploid gametes of the individual are formed. This implies that if homing conversion efficiency is 100%, the individual only produces gametes carrying the gene drive allele. In post-zygotic homing gene drives, homing occurs after the formation of the zygote, so that a zygote formed with a heterozygous combination of the gene drive and wild-type alleles will be homozygous for the gene drive. This difference affects the impact of selection, because in the prezygotic system, the gene drive can exist as a heterozygote, but in the post-zygotic system all gene drives should be homozygous. The most important model information is captured in the vector of gene drive and wild-type allele frequencies *P*, which is used to compute the matrix of diploid genotype frequencies, *G*, as the outer product of *P* with itself. The homing conversion, selection, and genetic drift alter the genotype frequencies that are modeled as detailed below, using sequential modules computing the matrix of their effect on each genotype and updating the genotype frequency.

**Table 1.** Variables definitions and distribution used in the Monte Carlo simulations.

Variable	Definition	Symbol	Distribution
Conversion	Rate at which a gene drive allele is able to convert a wild-type allele in heterozygous genotypes in 1 generation	*c_ij_*	U(0,1)
Resistance level	Conversion rate of gene drive on resistance allele	*R_j_*	U(0,1) × Conversion
			
Resistance frequency	Initial frequency of alleles resistant to gene drive conversion	$p_{j}^{R}$	U(0,0.4)
Exposure rate	Proportion of population exposed to selection acting negatively on gene drive heterozygous and homozygous genotypes	*e*	U(0,1)
Fitness cost	Selection coefficient of genotypes carrying a gene drive allele	*s_ij_*	U(0,1)
Dominance	Level of dominance of the gene drive allele on the fitness of heterozygous genotypes. 0 = recessive; 0.5 = additive; 1 = dominant	*d_ij_*	U(0,1)
Inbreeding	Reduction in frequency of heterozygous genotypes compared with the Hardy–Weinberg expectation	*F*	U(0,1)

### Gene drive homing conversion

The conversion module simulates the CRISPR-mediated homing conversion of a wild-type allele into a gene drive allele in heterozygous individuals as illustrated graphically in Bier (2022). Genotypes that are heterozygous for the gene drive allele have a probability to undergo conversion and become homozygous for the gene drive allele as:


(1) $$\Delta G_{ij}=x/n,\;\mathrm{where}\;x∼\mathrm{Binomial}(n,c_{ij}\times G_{ij}),$$

where *n* is the population size, *c_ij_* is the conversion efficiency of gene drive allele *i* on wild-type allele *j*, which defines the proportion of allele *i* that will be converted in allele *j* in heterozygous genotypes *ij* present in the population at frequency *G_ij_*. The proportion of wild-type alleles converted in heterozygous genotypes is subtracted from the frequency of wild-type alleles and added to that of the gene drive. For resistant alleles, the initial frequency $p_{j}^{R}$ was sampled from a uniform distribution, setting 0.4 as the upper limit to model them as minor alleles. Their conversion efficiency *c_ij_*, is scaled by a factor sampled from a uniform distribution. This resistance level *R_j_*, then replaces the conversion efficiency *c_ij_*. The allele frequencies are then recomputed so that the wild-type allele, resistance allele, and gene drive frequencies sum to 1.

### Selection

The selection module simulates the effect of selection on diploid genotypes acting against the gene drive that experiences a fitness cost when exposed to selection. It factors 3 variables: the fitness cost of the gene drive allele, which is modeled as an intrinsic genetic property; the exposure rate, representing the fraction of the population exposed to selection, which depends on the environment and management practices; and the dominance effect of the gene drive allele on the fitness of a genotype. Genotypes only carrying wild-type or resistant alleles have a fitness of 1. The fitness cost of a gene drive in a heterozygous genotype is affected by the dominance effect *d* of the gene drive allele, which ranges continuously from recessive (*d* = 0), to additive (*d* = 0.5), and to dominant (*d* = 1). For simplicity, we chose not to explore dominance values exceeding 1, which would represent cases of overdominance in the fitness cost of the gene drive, as there is then no theoretical upper boundary that would define a finite space for the dominance variable. Change in the frequency of a genotype in response to selection, whose intensity is determined by the exposure to selection, is proportional to the fitness of the genotype:


(2) $$\Delta G_{ij}=x/n,\;\mathrm{where}\;x∼\mathrm{Binomial}(e\times G_{ij}\times n,1-d_{ij}\times s_{ij}),$$

where *e* is the exposure to selection of the population (exposure rate hereafter), *G_ij_* is the genotype frequency, *n* is the population size, *d_ij_* is the dominance of the fitness cost, and *s_ij_* is the fitness cost of genotype *ij*. The full definition space of selection against the gene drive from 0 (neutral) to 1 (lethal) was explored to allow for extremely deleterious gene drives to be modeled as in the case of recessive lethal drives (Bull *et al*. 2019; Gomulkiewicz *et al*. 2021). The change in genotype frequency (*ΔG_ij_*) is computed and subtracted from its initial frequency. The *n* value is scaled by the percentage of the population exposed to the selection pressure. After selection, genotype frequencies are scaled to sum to 1.

### Genetic drift

Genetic drift is simulated by resampling each allele from a Binomial distribution and converting the outcome of a random draw divided by twice the population size as:


(3) $$p_{i}^{\prime }=x/2n,\;\mathrm{where}\;x∼\mathrm{Binomial}(2n,p_{i}),$$

where *p_i_*′ is the allele frequency of allele *i* after genetic drift, *n* is the population size, and *p_i_* is the initial allele frequency. A population size of 1,000,000 was kept constant over time in all simulations, modeling the effect of a replacement gene drive, aiming to substitute wild-type alleles for the gene drive.

### Diploid zygote formation

Zygote formation leading to diploid genotypes assumes Hardy–Weinberg equilibrium except for allowing a level of inbreeding *F*, so that genotype frequencies for every combination of alleles *i* and *j* among *N* are given by:


(4) $$G_{ii}=p_{i}^{2}+Fp_{i}p_{j};G_{ij}=2(1-F)p_{i}p_{j};G_{\;jj}=p_{j}^{2}+Fp_{i}p_{j}\;\mathrm{With}\;\overset{N}{\underset{i\neq j}{\sum }}\;G_{ii}+G_{ij}+G_{\;jj}=1$$

### Outcome classification

The outcomes were classified based on the maximum frequency, final frequency, and change in frequency over time of the gene drive allele after 500 generations (Fig. 1c). The maximum frequency is the highest frequency the gene drive allele reaches over the course of a simulation. The final frequency is the frequency of the gene drive allele in the last generation. The change in frequency over time, referred to as gradient hereafter, is the change in gene drive allele frequency over the last 20 generations. A simulation was classified as fixation if the final frequency is >0.90; loss if the maximum frequency is <0.10; temporary if the maximum frequency is >0.30 and a final frequency <0.10; and equilibrium if the final frequency is between 0.10 and 0.90 with the absolute value of the gradient <0.0001 in the last 20 generations (Fig. 1c). Simulations that did not meet these criteria remained unclassified and were not analyzed further. Within these 4 gene drive outcomes, the allele frequency dynamics of the gene drive were analyzed using 3 metrics: the maximum frequency, the time to maximum frequency, and the final frequency.

### Implementation

The modular structure of the model allows for the steps to be reordered to reflect different gene drive systems. This enables the modeling of both pre- and post-zygotic gene drive systems. In our post-zygotic gene drive model, conversion occurs before selection (Fig. 1a), whilst in a prezygotic system, conversion occurs after selection. Our stochastic model was validated against the results from published deterministic models (Supplementary Figs. 1–3; Unckless *et al*. 2015; Drury *et al*. 2017; Rode *et al*. 2019). Stochastic variation in the simulations was introduced through pseudorandom numerical sampling, and its magnitude was primarily determined by the population size.

The model is available at adaptive-evolution.biosciences.unimelb.edu.au/SIEGE/through a graphic user interface. A user-friendly implementation of the model intends to provide a quick and direct way to run our model in order to understand how different properties of a gene drive interact to influence its frequency dynamics. Scripts to deploy and run the model locally can be sourced from https://github.com/bencamm001/interactive_gene_drive. A sensitivity analysis on the model variables was conducted using a multivariate Monte Carlo simulation framework. Variables in Table 1 were randomly sampled from a uniform distribution to explore the full definition space for a variable without a priori knowledge and the model was initially run 60,000 times to estimate the outcome frequencies (Table 2). To conduct further analysis with equal sample size across outcomes, another set of simulations was conducted, randomly sampling variables until 60,000 simulations of each outcome were observed. This was done to dissect the effect of all variable combinations across all outcomes with even power.

**Table 2.** Frequency of outcomes among 60,000 pre- and post-zygotic simulations.

Outcome	Prezyogtic outcome frequency (%)	Post-zygotic outcome frequency (%)
Loss	51.53	58.17
Fixation	33.65	32.02
Temporary	6.42	4.75
Equilibrium	1.34	0.42
Total	92.94	95.37

### Statistical analysis

Simulations were analyzed using 2 complementary approaches to measure the importance of variables in determining a gene drive outcome. Principal component analysis (PCA) was used as a parametric method of dimensionality reduction suitable when analyzing correlated variables and relatively insensitive to noise. Random forest (RF) was used as this ensemble decision tree approach applies particularly well to classification problems where variable response is nonlinear, with a low sensitivity to overfitting. PCA was conducted using the Julia\MultivariateStats package (v0.8.0) on a down-sampled set of 2,500 simulations from each outcome to ease computation and visualization, using all 7 variables listed in Table 1. Covariance ellipses for each outcome are the elliptic contour line of a Gaussian density function with equal variance to that observed in the data, drawn using the *covellipse* function from the StatsPlots package. This was done to qualify the variation of each variable that determines the outcome. RF regression was used to measure the importance of all the variables in a random set of 40,000 simulations (10,000 from each outcome) from the sensitivity analysis using the R\randomForest package (v4.6.14). Variable importance was measured in terms of the mean decrease in mean square error upon noninclusion of each variable for determining a given outcome. RF models were fitted to the data by drawing 500 trees using 3 variables for each tree.

## Results

### Frequency of the different outcomes

A very high proportion of the 60,000 initial Monte Carlo simulations were classified as either fixation, loss, temporary, or equilibrium (92.94% for the prezygotic and 95.37% for the post-zygotic model). Under both models, equilibrium was the rarest outcome, only found in 1.34% of prezygotic and 0.42% of post-zygotic simulations. The next rarest outcome was temporary, represented by 6.42% of prezygotic simulations, and by 4.75% of post-zygotic simulations (Table 2). The uncategorized simulations corresponded to cases where the gene drive maximum frequency ranged between 0.1 and 0.3 (neither loss nor temporary) or where the maximum frequency exceeded 0.1 but without plateauing (neither fixation nor equilibrium).

**Fig. 2.**
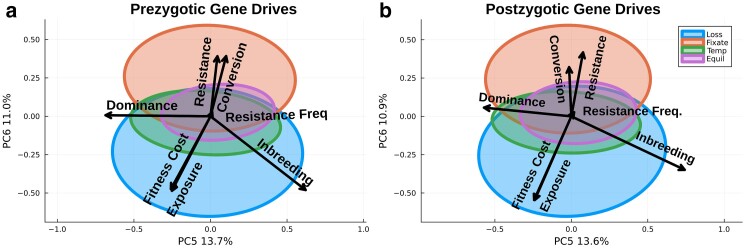
PCA of the post-zygotic Monte Carlo simulations based on the initial variables described in Table 1. PC5 and PC6 are used for a clear visualization of outcomes as all PCs explained a similar proportion of the variance. Covariance ellipses shown for clarity due to overlapping groups.

PCA was used to assess how variables individually contributed to each outcome and how independent the different outcomes were over the variable space explored (Fig. 2a for prezygotic model and Fig. 2b for post-zygotic model). The analysis of prezygotic gene drive simulations for each outcome showed that no single PC strongly distinguished outcomes, which was qualitatively similar in post-zygotic simulations. This suggested that no single variable was able to drive a specific outcome. Instead, the variable space permitted by the different outcomes largely overlapped. Predictably, loss and fixation were the most distinct outcomes, with temporary and equilibrium representing intermediates between these. The variables with the strongest contribution to fixation were the conversion efficiency and gene drive resistance level and frequency, while the fitness cost of the gene drive, exposure to selection, and inbreeding level differentiated loss from the other outcomes. The dominance of the fitness cost of the gene drive differentiated temporary from the equilibrium outcome. Interestingly, the resistance frequency had minimal contribution to outcome determination.

Variable importance measured through RF models was consistent with the PCA. Dominance was the most important variable in determining the equilibrium outcome, with its noninclusion in the model increasing the mean squared error by 365.16 (Table 3). Selection pressure (the product of fitness cost and exposure rate) was important for all outcomes. Consistent with the PCA results, resistance frequency was not overly important in driving outcomes; however, among outcomes, it was most important in determining the temporary outcome. Both the PCA and RF results suggested that every variable tested could be used to influence the outcome, but it is likely that variables will need to be combined to efficiently determine the fate of a gene drive. Because the variables effect and importance on outcome determination were qualitatively consistent between pre- and post-zygotic gene drives, further analysis was restricted to prezygotic gene drives, which is the mode of action of the most extensively experimentally validated CRISPR-based homing gene drive (Kyrou *et al*. 2018; Hammond *et al*. 2021).

**Table 3.** Variable importance on the outcome determination for pre- and post-zygotic gene drive in MC simulations measured in terms of mean squared error upon noninclusion.

	Prezygotic					Post-zygotic				
	Loss	Fixate	Temporary	Equilibrium	All outcomes	Loss	Fixate	Temporary	Equilibrium	All outcomes
Conversion efficiency	198.7	39.21	184.7	40.36	924.63	203.01	42.71	177.48	36.45	920.53
Resistance level	62.79	286.66	319.04	288.41	1,566.99	69.02	307.75	339.3	311.6	1,600.25
Resistance frequency	10.01	5.21	42	16.74	311.41	7.99	2.38	40.69	19.35	299.41
Selection pressure*^a^*	278.6	405.15	329.01	342.2	2,080.59	277.22	385.46	320.89	337.08	2,013.77
Dominance	105.9	29.25	106.25	351.05	1,036.07	105.02	42.58	104.58	365.16	1,055.45
Inbreeding	311.5	145.37	269.55	325.73	1,579.16	297.73	151.84	245.1	312.35	1,609.78

^*a*^Selection pressure is the product of the gene drive’s fitness cost times exposure to selection.

**Fig. 3.**
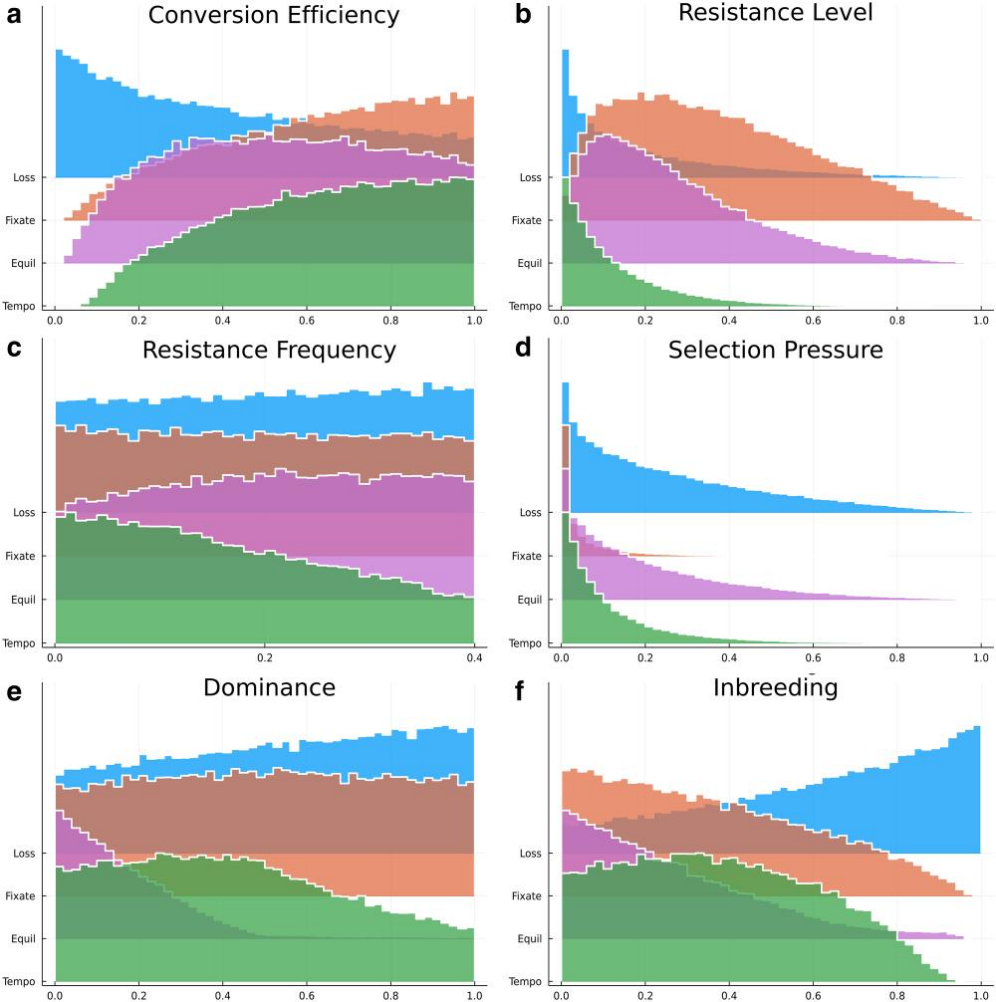
Distribution of the variables within each outcome. Resistance and selection pressure (the product of selection coefficient and exposure rate) show skewed distributions as they are the product of 2 uniform distributions.

The distribution of variables within outcomes was used to assess the level of constraint on the value a given variable could take (Fig. 3); a narrow distribution indicated a limited space over which a variable can vary without modifying the outcome of the gene drive. Every variable except resistance frequency showed a constrained distribution for at least 1 outcome, with a maximum density value centered toward 0 or 1. Conversion efficiency was the most negative for the loss outcome and positive for the fixation outcome. Equilibrium and temporary outcomes were mostly driven by high values of conversion efficiency but not as strongly as fixation (Fig. 3a). The loss and temporary outcomes were driven by low resistance levels, compared with fixation and equilibrium where the resistance level could take higher values (Fig. 3b). Selection pressure (the product of fitness cost and exposure rate; Fig. 3d) showed the strongest skew in distribution for the fixation outcome, with the highest density near zero, while temporary outcomes tolerated a slightly broader range of selection pressures; equilibrium and loss outcomes tolerated the widest range of selection pressures. Hence, a temporary outcome would be best achieved through the combination of strong resistance and weak selection pressure.

The likelihood of an equilibrium outcome dramatically increased when dominance values were <∼0.5, which corresponds to a recessive fitness cost for the gene drive allele (Fig. 3e). Equilibrium outcomes were less likely to occur when inbreeding was high. Fixation and temporary outcomes tolerated a wider range of inbreeding values, while loss occurred most frequently when inbreeding was high (Fig. 3f). A moderately low resistance level also promoted equilibrium (Fig. 3b). Designing a gene drive for an equilibrium outcome would thus require low levels of inbreeding and dominance, to which a nonnull selection pressure should be added to escape a fixation or temporary outcome. There was a narrower range of selection for post-zygotic simulations (Supplementary Fig. 4). This level of constraint explains the rarity of the equilibrium outcome across our simulations.

### Gene drive frequency dynamics within outcomes

**Fig. 4.**
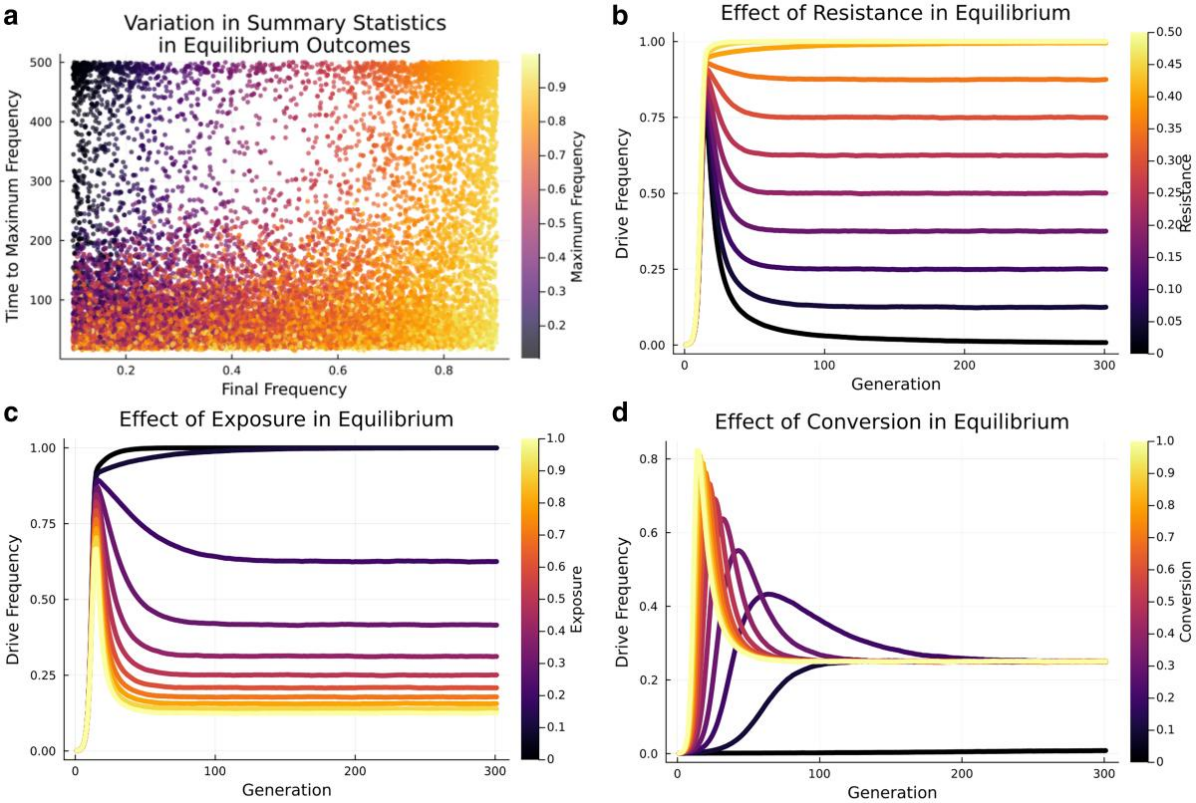
Effect of variables within equilibrium outcomes. a) Variation in final frequency, maximum frequency and time to maximum frequency. b) Effect of wild-type alleles resistance levels on gene drive frequency. c) Effect of exposure rate to selection against gene drive fitness cost. d) Effect of wild-type conversion efficiency. Variables if not the focus of the analysis was set to: conversion efficiency = 0.95, selection coefficient = 0.8, exposure rate = 0.5, resistance level = 0.1, resistance frequency = 0.1, degree of dominance = 0.0, and inbreeding = 0.0.

The equilibrium outcome is complex to achieve owing to the broad variation in the gene drive maximum frequency and time to maximum frequency defining this outcome. Within equilibrium outcomes, maximum frequency ranged from 0.1 to 0.99 and time to maximum frequency ranged from 16 to 500 generations (Fig. 4a). The simulations in which the time to maximum frequency tended toward 500 generations were characterized by high levels of inbreeding. Inbreeding slowed the gene drive frequency increase to the point of equilibrium, so that the final frequency was also the maximum frequency. The final frequency of a gene drive at equilibrium was most strongly determined by the resistance and the selection pressure (Table 4). The final frequency was positively correlated to the resistance level (Fig. 4b) and negatively to the exposure rate (Fig. 4c). These 2 variables thus partly determined the equilibrium frequency of a gene drive. In turn, the time to equilibrium frequency depended on the conversion efficiency, with lower efficiency slowing the gene drive (Fig. 4d). Interestingly, the resistance frequency, which had minimal importance in determining the outcome or the final frequency of the gene drive, had a strong effect on the time to maximum frequency (Table 4).

**Table 4.** Effect of model variables on prezygotic gene drive frequency within specific outcomes.

	Equilibrium outcomes						Temporary outcomes					
	Time to maximum frequency			Final frequency			Time to maximum frequency			Maximum frequency		
	Regression coefficient	Std. error	Pr(>|t|)	Regression coefficient	Std. error	Pr(>|t|)	Regression coefficient	Std. error	Pr(>|t|)	Regression coefficient	Std. error	Pr(>|t|)
Conversion efficiency	−255.68	2.06	<1e−99	0.014	0.004	0.0007	−181.33	0.76	<1e−99	0.29	0.0024	<1e-99
Resistance level	116.97	4.35	<1e−99	0.92	0.0085	<1e−99	158.56	1.63	<1e−99	0.14	0.0052	<1e-99
Resistance frequency	148.59	3.87	<1e−99	−0.02	0.0075	0.0037	−13.03	1.45	<1e−18	−1.18	0.0047	<1e-99
Fitness cost	−73.03	2.62	<1e−99	−0.59	0.0051	<1e−99	6.58	0.91	<1e−12	−0.41	0.0029	<1e-99
Exposure	−73.32	2.63	<1e−99	−0.6	0.0051	<1e−99	7.46	0.91	<1e−15	−0.41	0.0029	<1e-99
Dominance	210.68	2.95	<1e−99	0.18	0.0058	<1e−99	42.63	0.6	<1e−99	0.01	0.0019	<1e-99
Inbreeding	247.04	3.08	<1e−99	−0.4	0.006	<1e−99	153.98	0.94	<1e−99	−0.37	0.003	<1e-99

**Fig. 5.**
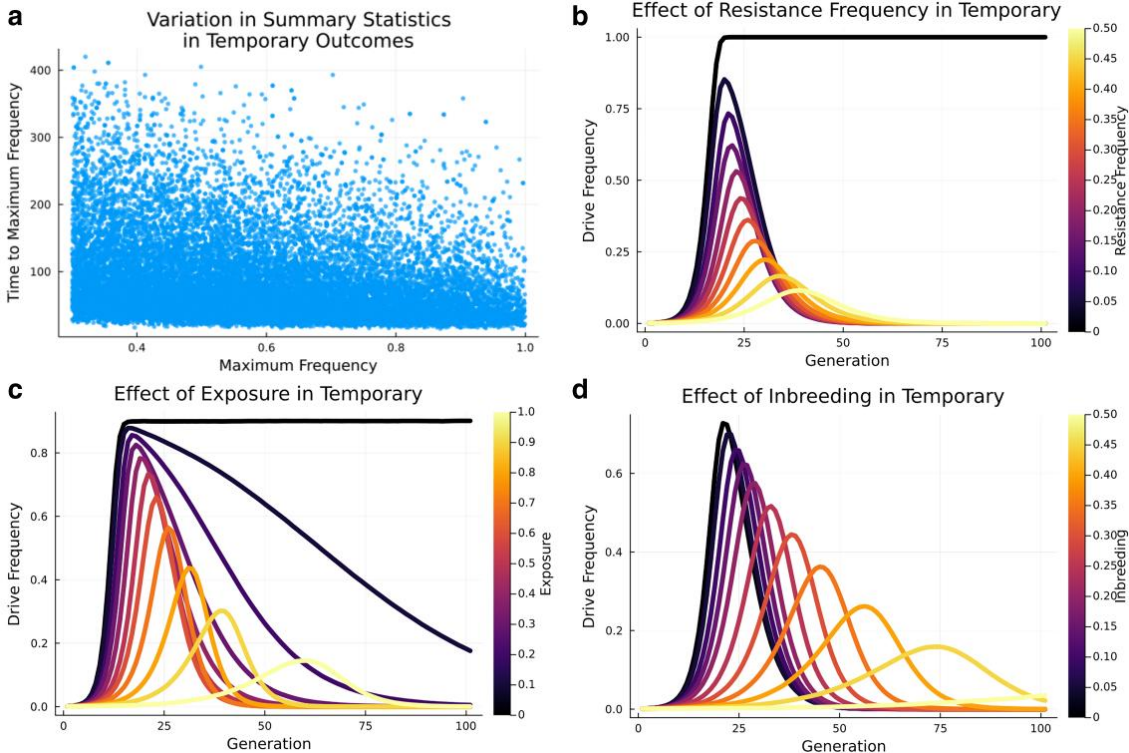
Effect of variables within temporary outcomes. a) Variation in maximum frequency and time to maximum frequency. b) Effect of the resistance frequency. c) Effect of exposure rate to selection against gene drive fitness cost. d) The effect of inbreeding. Variables if not the focus of the analysis were set to: conversion efficiency = 0.95, selection coefficient = 0.8, exposure rate = 0.5, resistance level = 0.0, resistance frequency = 0.1, degree of dominance = 0.5, and inbreeding = 0.0.

As with equilibrium, temporary outcomes could be achieved for a broad range of maximum frequencies and times to maximum frequency. Among the prezygotic simulations leading to temporary outcome, maximum frequencies ranged from 0.30 to 0.99 and the time to maximum frequency ranged from 17 generations to 474 generations (Fig. 5a). The 2 variables showing the strongest effect on the maximum frequency within the set of simulations reaching a temporary outcome were the resistance frequency and the selection pressure (Table 4). The initial frequency of resistant alleles was negatively correlated to the maximum frequency of the gene drive (Fig. 5b). This was due to the fact that the initial frequency of the resistance alleles, if remaining constant over time in the population, is the ceiling of the gene drive frequency. When the resistance frequency is zero, the gene drive fixates; when exposure of the fitness cost to selection increases, the maximum frequency decreases (Fig. 5c). Contrary to what was found for equilibrium outcomes, the time to maximum frequency in temporary outcomes was most strongly regulated by the resistance level, followed by conversion efficiency (Table 4). Finally, increasing inbreeding and exposure rate both increased the time to maximum frequency, with higher levels of inbreeding also decreasing the overall maximum frequency of temporary gene drives (Fig. 5d). Variable effects for post-zygotic simulations are shown in Supplementary Fig. 4 and Supplementary Table 1.

### Switching from an equilibrium to a temporary outcome

**Fig. 6.**
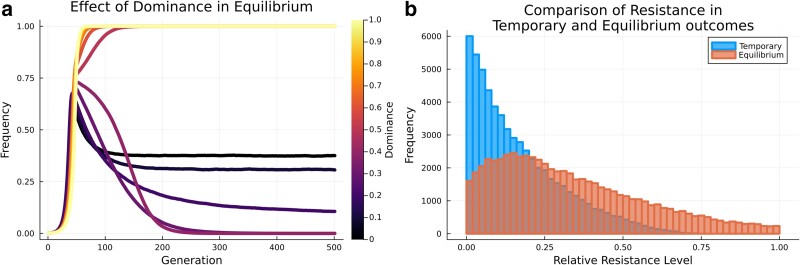
a) Effect of the level of dominance in promoting a switch between gene drive fixation, temporary and equilibrium outcomes. Variables if not the focus of the analysis were set to: conversion efficiency = 0.95, selection coefficient = 0.8, exposure rate = 0.5, resistance level = 0.3, resistance frequency = 0.1, and inbreeding = 0.0. b) The relative resistance conversion efficiency as a fraction of the conversion efficiency ($\frac{\mathrm{conversion}}{\mathrm{resistance}}$) between temporary and equilibrium outcomes.

While we have shown how variables are important for determining an outcome, we also want to know how changing a variable can shift 1 outcome to another. For a post-zygotic gene drive with specific combinations of variable values, changing the level of dominance of the gene drive fitness cost was able to shift the fate of a gene drive from equilibrium to temporary to fixation (Fig. 6a). Overall, dominance was thus pivotal in determining the final equilibrium frequency of a gene drive, from fixation to equilibrium to temporary (Fig. 4b and c). However, the determining factor between equilibrium and temporary was not the absolute value of conversion efficiency and resistance level but the ratio between them. Temporary outcomes generally showed a greater difference between the conversion efficiency and resistance, while equilibrium outcomes tolerated a wider range (Fig. 6b). This suggests that if a temporary outcome is intended, the ratio between conversion efficiency and resistance should approach zero, while for equilibrium outcomes it should be close to ∼0.2.

## Discussion

Gene drives are complex systems influenced by multiple factors, some controllable by design, some imposed by the biology of the species or population targeted. Our work focused on the conditions able to promote intermediate equilibrium and temporary outcomes. While less frequent, they are particularly relevant for population management as they will not lead to irreversible loss of genetic diversity. These outcomes would be safer for early gene drive releases (Esvelt and Gemmell 2017; Noble *et al*. 2019) but required a combination of factors in our model. We identified specific combinations of values for the variables tested that could lead to a given outcome but also affect or alter the frequency dynamics of a gene drive to switch outcomes.

### Balance between conversion efficiency and resistance for intermediate outcomes

One of the foundational predictions of the Wright–Fisher model is that alleles evolving neutrally and only undergoing random frequency change due to drift are doomed to either fixate or get lost, particularly in small populations. For a homing gene drive to remain at intermediate frequency, the homing conversion efficiency needs to be balanced by selection (Noble *et al*. 2019). Temporary outcomes can occur when the conversion efficiency differs between alleles in the population, which can be engineered by the design of the gene drive. This can occur if the CRISPR elements lose their ability to convert other alleles into gene drives over time. This is likely to occur as no purifying selection is expected to maintain the integrity of the CRISPR elements. A temporary outcome can be achieved through engineered efficiency decay, as proposed in daisy chain (Noble *et al*. 2019) or split drive systems (Akbari *et al*. 2015; Edgington *et al*. 2020; Kandul *et al*. 2020). Alternatively, mutations in the gRNA target sequence of the gene drive may inhibit its function (Hsu *et al*. 2013; Unckless *et al*. 2017; Price *et al*. 2020; Grewelle et al., 2022). Polymorphic alleles may be more or less complementary to the gRNA, leading to the degree of quantitative resistance explored in our model (Willis and Burt 2021). Even with conversion efficiencies remaining constant, gene drive alleles convert wild-type alleles but not resistant ones, mechanically increasing resistance frequency. The resistance frequency thus sets which fraction of the alleles is immune to the gene drive, and hence its maximum frequency. Next, as gene drives often aim to reduce the carrier’s fitness, conversion-resistant alleles are expected to have a higher fitness and increase in frequency relative to the gene drive, causing the gene drive frequency to decrease until lost (Drury *et al*. 2017).

Equilibrium relies on the balance between conversion efficiency and selection against the gene drive (Unckless *et al*. 2015; Rode *et al*. 2019), with the increase in gene drive frequency due to conversion matching the decrease in frequency due to its fitness cost. In the absence of selection against the gene drive, equilibrium can be reached after all susceptible wild-type alleles have been converted, leaving only fully resistant alleles behind. We show that equilibrium is more likely under moderately efficient conversion. This is promising as conversion efficiency is unlikely to be 100% in natural biological systems (Hsu *et al*. 2013; Prowse *et al*. 2017; Champer, Oh, *et al*. 2020; Verkuijl *et al*. 2022; Du *et al*. 2024). A gene drive designed to reach a stable, low frequency equilibrium would not fixate in the population and have less risk of escaping to neighboring populations through gene flow. Recent work has also shown that excessively efficient suppression gene drives are likely to empty the niche of the population targeted, leaving the opportunity for wild-type, undesired genotypes to recolonize, hence defeating the very purpose of the gene drive (Champer *et al*. 2021; Paril and Phillips 2022). A suppression gene drive designed to target the malaria agent *Plasmodium falciparum* and reduce its fecundity within the host could be designed to reach equilibrium (North *et al*. 2020). This would not cause the extinction of the parasite species but would reduce the population size and hence transmission and the overall incidence of malaria (Tachibana *et al*. 2022; Wang *et al*. 2022).

### Constraints and feature importance for designing gene drives

Consistent with previous studies, we determined that the variable space leading to intermediate outcomes is narrow (Eckhoff *et al*. 2017; Rode *et al*. 2019), with the strongest driver being the dominance of the fitness cost. RF models identified rather accurately which aspect of the gene drive design to focus on to ensure a desired outcome. Ideally, an equilibrium gene drive will have a recessive fitness cost and target an outbreeding population. However, in practice, the level of control of each of the variables tested here varies greatly. The molecular properties of a gene drive can be controlled by design, some population features can be managed, but others are intrinsic to the target population. For example, the level of inbreeding of the population is unlikely to be controlled, but the conversion efficiency, fitness cost, and exposure rate could be adjusted by design.

In the previous section, we discussed the critical importance of the balance between resistance level and conversion efficiency balance for controlling gene drive outcomes. Resistance to a gene drive can rise from multiple sources, whether standing genetic variation, de novo mutation, or nonhomologous end joining (NHEJ) after a failed gene drive homing (Price *et al*. 2020). Resistance to homing-based gene drives can be caused by mutations stopping the directed endonuclease protein from reaching its target. The likelihood of a mutation causing immunity can be limited by the use of Cas endonucleases edited to be protospacer adjacent motif (PAM)-promiscuous (Hu *et al*. 2018), or cut sites distal to the PAM site that are less likely to cause NHEJ (Paul and Montoya 2020). Gene drive resistance occurring through NHEJ after CRISPR cleavage can also be limited through gRNA multiplexing (Champer, Oh, *et al*. 2020). In a CRISPR-based gene drive, the similarity between the gRNA and its target site determines the cleavage efficiency, and therefore the conversion efficiency (Hsu *et al*. 2013). Hence, the resistance frequency can be controlled through gRNA target site choice in the same way the resistance level is controlled. Polymorphism in the gRNA target site can be leveraged to engineer gene drives to be temporary or to reach equilibrium. Future models will improve in specificity and accuracy by incorporating information about the genetic diversity at gRNA target sites and its effect on the progression of a gene drive.

We also showed that, as far as all wild-type alleles are not fully immune to gene drive homing, it is possible to manage resistance. Our model explored the effect of quantitative levels of resistance to a gene drive, which is rarely considered. Also, if the resistance allele has a low frequency, the gene drive is still able to increase in frequency up to a point of equilibrium with the resistant alleles. At this point, if the gene drive frequency exceeds the equilibrium threshold, it will fixate; if it is below, it will become a temporary drive and be lost (Unckless *et al*. 2015; Greenbaum *et al*. 2021). It is through this unstable equilibrium that a gene drive can overcome highly resistant alleles.

### Indirect control through exposure to natural selection

The frequency of equilibrium can be fine-tuned through resistance and selection pressure. The selection pressure can be controlled, either by designing a gene drive with a specific fitness cost or by varying the exposure to selection through population management. Gene drives expressing a toxin are likely to be dominant, and their cost will depend on the effect of the toxin (Champer, Kim, *et al*. 2020). For gene drives spreading susceptibility to a xenobiotic (Wedell *et al*. 2019; Bier 2022), the response to selection will depend on the rate of exposure to selection. This exposure rate can be anthropogenically managed through pesticide application, for instance (Carrière *et al*. 2012). We have shown the pivotal importance of the dominance of the fitness cost for determining the outcome of a gene drive. For gene drives targeting essential loci for which some level of genetic redundancy is expected, the fitness effect of the gene drive is likely to be more recessive. The way selection pressure is managed during the spread of a gene drive will thus be critical to ensure the gene drive acts as intended. While our model describes the behavior of a replacement gene drive, analyzing the differential selection between genotypes carrying or not a gene drive allele, dominant or not, is likely to have an effect on the population demography and hence induce some level of suppression. Our findings on the effect of selection are likely to hold in the context of a suppression gene drive at the early phase of a release while the population is still large. As the population size becomes small, modeling stochastic processes such as drift and mating will be affected, and this avenue should be the scope of future modeling efforts.

We found that gene drives are more likely to reach equilibrium in a population when acting prezygotically and when their fitness cost is recessive. Prezygotic drives were also more likely to be temporary compared with post-zygotic drives. The prezygotic gene drive system is more tolerant of high fitness costs, which may be useful for suppression gene drives, which are likely to incur a high fitness cost. A post-zygotic drive is unable to spread when selection against it is too strong, even under a recessive genetic architecture (Unckless *et al*. 2015). However, selection pressure is rarely homogeneous over time and space (Lenormand 2002; Carrière *et al*. 2012). Factoring an exposure rate in our model catered to the potential heterogeneity of selection that could be induced by, for example, a selection regime where a location where half the population is treated with a pesticide and half is not. Modeling the ubiquitous application of a pesticide that kills 50% of the population is the same as applying a 100% lethal pesticide on half of a population. However, in a spatially explicit model, it may show changes in population structure due to the fitness differential. Most models have so far assumed that selection pressure is constant and ubiquitous when in most natural systems, selection varies over space and time. Relaxing selection through management practices would allow the gene drive to spread in spite of a high selection pressure (Green *et al*. 2020). This allows for more accurate modeling of a gene drive spreading susceptibility to a xenobiotic, such as herbicide resistance in weeds.

## Conclusion

Building confidence in the fact that gene drives will act as intended is vital. There have been too many dreadful examples of biocontrol measures becoming biosecurity issues (Blossey and Notzold 1995; Shine 2010; Schulz *et al*. 2019). Models provide guidelines and driving principles but can also be leveraged for the in silico stress testing of new tools. Our model provides a generic tool that can be applied broadly. It is currently species-agnostic and has no spatial component, but future developments building on the modular design could accommodate more specificity. Incorporating more specific information about gene drives’ biology, such as how genetic variation within the gRNA target site may affect the conversion efficiency, is the next step considered. The effect of migration is hugely important to gene drives (Dhole *et al*. 2018; Greenbaum *et al*. 2021) and has not been included in this study despite being implemented in our modeling framework. Our work has explored how to design a gene drive that will achieve the intended outcome, but also for building confidence about our understanding of the frequency dynamics of a gene drive. This will aid in the development and design of future gene drives to maximize the likelihood that they will act as intended.

## Supplementary Material

jkae300_Supplementary_Data

## Data Availability

Scripts for running the model are available at https://github.com/bencamm001/interactive_gene_drive, and can be run online through a graphic user interface at http://adaptive-evolution.biosciences.unimelb.edu.au/SIEGE/. Supplemental material available at G3 online.
