# Supplementary material for: Controlling the frequency dynamics of homing gene drives for intermediate outcomes
Source: G3 (Bethesda). 2024 Dec 19;15(2):jkae300. doi: 10.1093/g3journal/jkae300 (PMC11797013; doi:10.1093/g3journal/jkae300)
Supplement: jkae300_Supplementary_Data [file jkae300_supplementary_data.pdf]

**- Supplementary Information -**

# Controlling the frequency dynamics of homing gene drives for intermediate outcomes

Benjamin J. Camm and Alexandre Fournier-Level

## Supplementary Figures

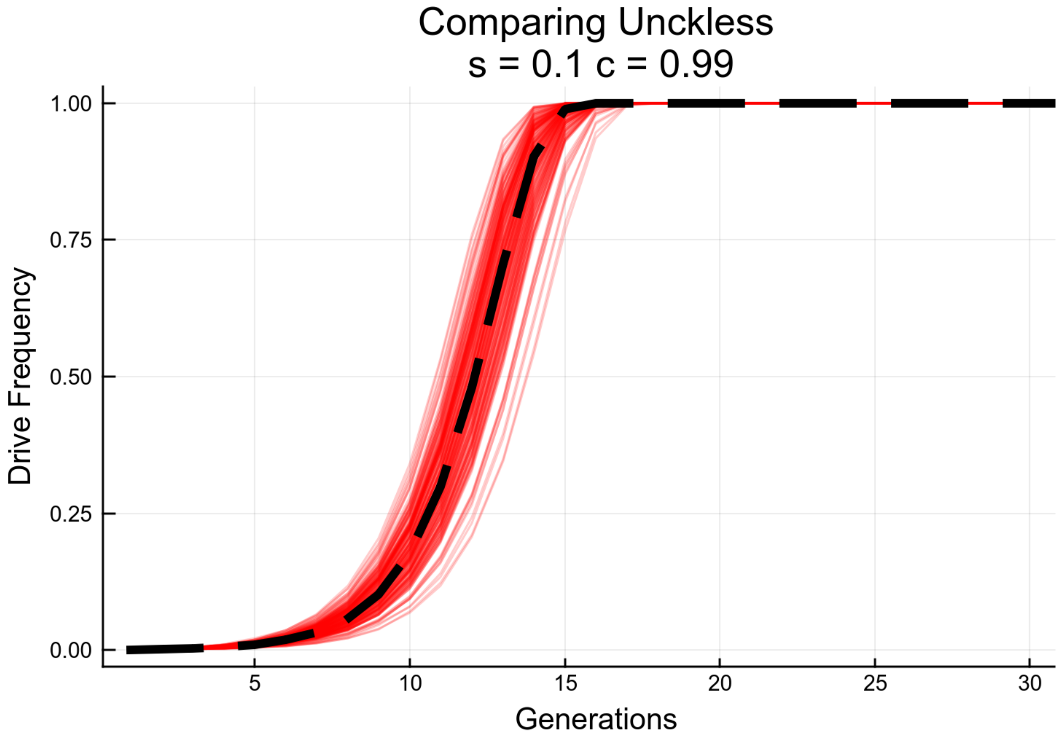


**Supplementary Figure S1:** Comparison of the stochastic model to Unckless et al. 2015's post-zygotic model. Using identical parameters, the dashed black line represents Unckless' model and the red lines show multiple simulations of our model.

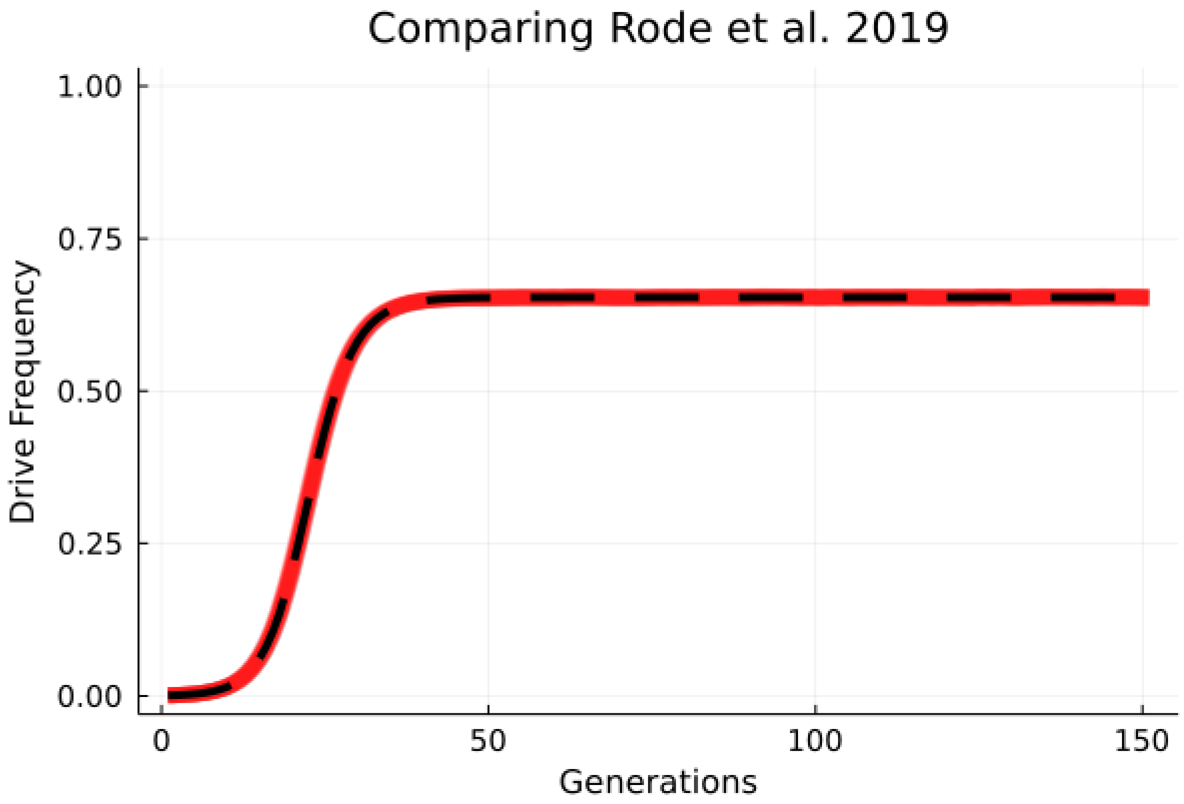


**Supplementary Figure S2:** Comparison of the stochastic model to Rode et al. 2019's pre-zygotic model. Using identical parameters, the dashed black line represents Rode's model and the red lines show multiple simulations of our model. Rode's model depicts a pre-zygotic model, hence our model was set accordingly (see Materials and Methods and Fig. 1a). In this comparison, conversion efficiency is 0.85, selection coefficient is 0.9 and dominance is 0.2.

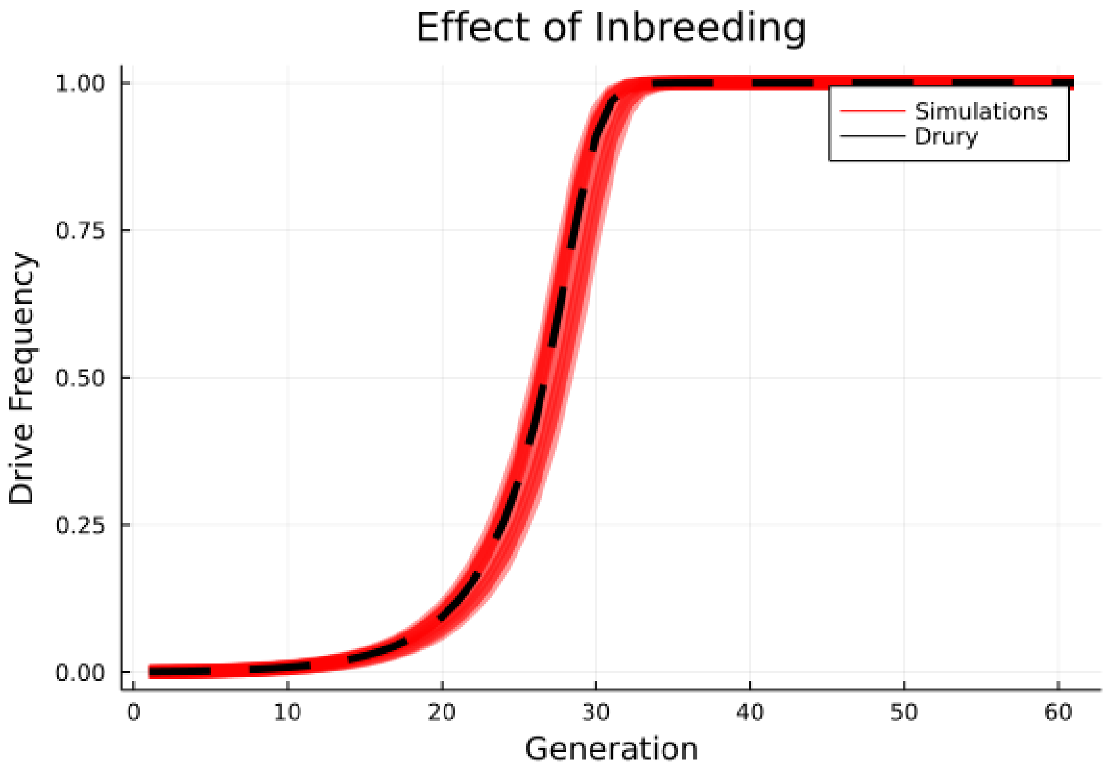


**Supplementary Figure S3:** Comparison of model to Drury et al. 2017's model (corrected). Using identical parameters, the dashed black line represents Drury's corrected model, and the red lines show multiple simulations of our model. For both models the conversion efficiency is 0.9, the selection coefficient is 0.3, dominance of 0.98 and inbreeding of 0.1, with no resistance alleles.

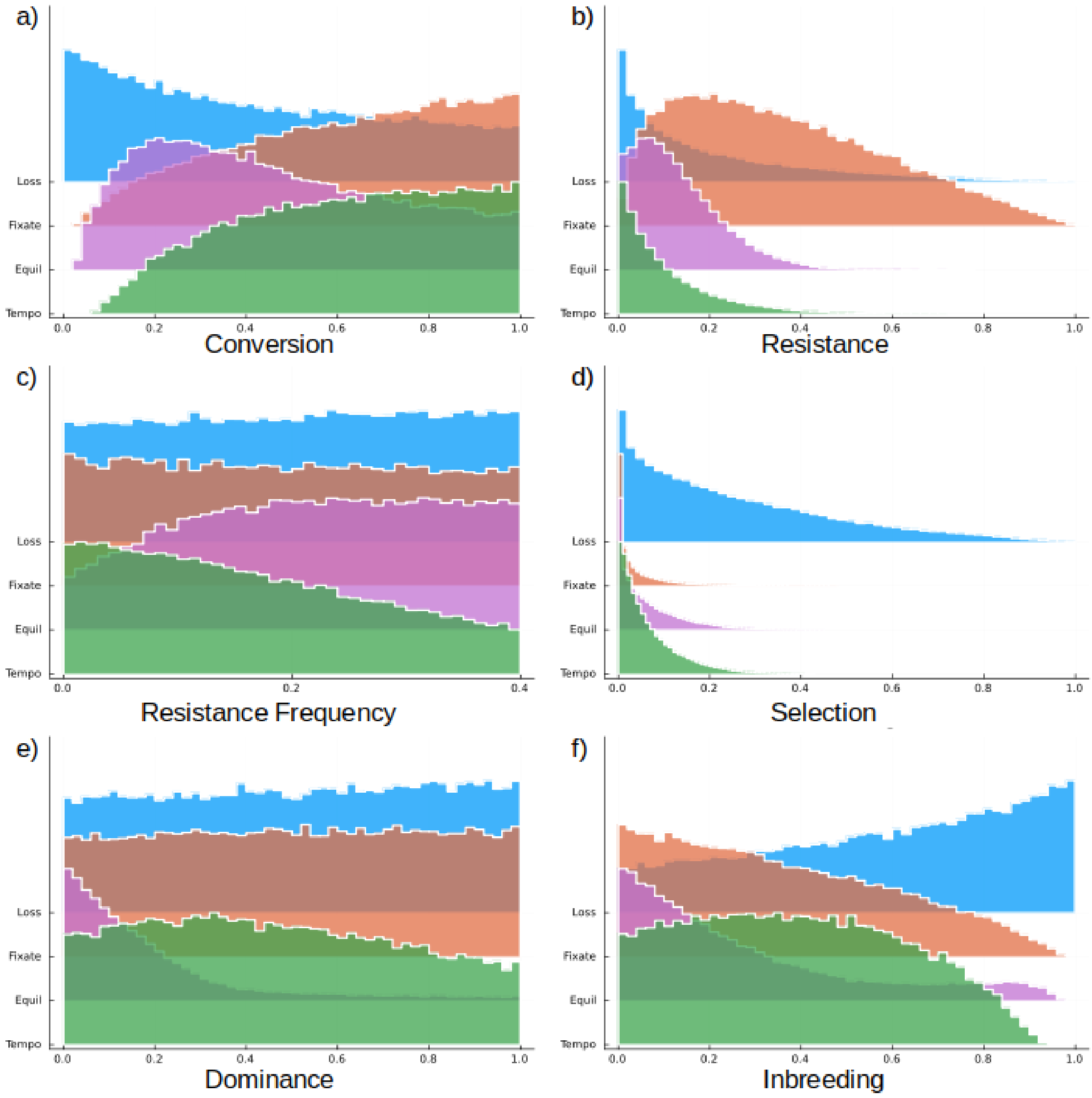


**Supplementary Figure S4.:**Variable distributions under a post-zygotic system.

| | Equilibrium outcomes | | | | | | Temporary outcomes | | | | | |
|---|---|---|---|---|---|---|---|---|---|---|---|---|
| | Time to Maximum frequency | | | Final frequency | | | Time to Maximum frequency | | | Maximum frequency | | |
| | Regression coefficient | Std. Error | Pr(>\|t\|) | Regression coefficient | Std. Error | Pr(>\|t\|) | Regression coefficient | Std. Error | Pr(>\|t\|) | Regression coefficient | Std. Error | Pr(>\|t\|) |
| Conversion | -276.72 | 2.3 | <1e-99 | -0.0203 | 0.0038 | <1e-7 | -195.92 | 0.79 | <1e-99 | 0.216 | 0.00247 | <1e-99 |
| Resistance level | 370.28 | 8.1 | <1e-99 | 0.6196 | 0.0134 | <1e-99 | 258.03 | 2.01 | <1e-99 | -0.00772 | 0.00632 | 0.222 |
| Resistance frequency | 302.67 | 5.04 | <1e-99 | -0.1307 | 0.0083 | <1e-55 | -29.97 | 1.56 | <1e-81 | -1.22 | 0.00489 | <1e-99 |
| Fitness cost | -78.64 | 3.39 | <1e-99 | -0.4136 | 0.0056 | <1e-99 | 15.55 | 1.07 | <1e-47 | -0.339 | 0.00352 | <1e-99 |
| Exposure | -77.03 | 3.4 | <1e-99 | -0.4088 | 0.0056 | <1e-99 | 15.94 | 1.07 | <1e-49 | -0.338 | 0.00336 | <1e-99 |
| Dominance | 170.1 | 3.03 | <1e-99 | 0.1907 | 0.005 | <1e-99 | 18.94 | 0.6 | <1e-99 | 0.055 | 0.00189 | <1e-99 |
| Inbreeding | 240.09 | 3.12 | <1e-99 | 0.0215 | 0.0051 | <1e-4 | 147.17 | 0.92 | <1e-99 | -0.229 | 0.00289 | <1e-99 |

**Supplementary Table 1**. Linear regression of variables' effect on summary statistics in postzygotic model.
